# Sâkipakâwin: Assessing Indigenous Cancer Supports in Saskatchewan Using a Strength-Based Approach

**DOI:** 10.3390/curroncol29010012

**Published:** 2021-12-28

**Authors:** Stephanie Witham, Tracey Carr, Andreea Badea, Meaghan Ryan, Lorena Stringer, Leonzo Barreno, Gary Groot

**Affiliations:** 1 Department of Community Health and Epidemiology, University of Saskatchewan, Saskatoon, SK S7N 5E5, Canada; stephanie.witham@usask.ca (S.W.); tracey.carr@usask.ca (T.C.); andreea.badea@usask.ca (A.B.); 2 Department of Surgery, University of Saskatchewan, Saskatoon, SK S7N 5E5, Canada; mhr849@mail.usask.ca; 3 College of Medicine, University of Saskatchewan, Saskatoon, SK S7N 5E5, Canada; lms790@mail.usask.ca; 4 Department of Sociology and Anthropology, Mount Royal University, Calgary, AB T3E 6K6, Canada; lbarreno@mtroyal.ca

**Keywords:** Indigenous, cancer supports, health disparities, needs assessment, oncology, strength-based approach

## Abstract

Given that the health care system for Indigenous people tends to be complex, fragmented, and multi-jurisdictional, their cancer experiences may be especially difficult. This needs assessment study examined system-level barriers and community strengths regarding cancer care experiences of Indigenous people in Saskatchewan. Guided by an advisory committee including Indigenous patient and family partners, we conducted key informant interviews with senior Saskatchewan health care administrators and Indigenous leaders to identify supports and barriers. A sharing circle with patients, survivors, and family members was used to gather cancer journey experiences from Indigenous communities from northern Saskatchewan. Analyses were presented to the committee for recommendations. Key informants identified cancer support barriers including access to care, coordination of care, a lack of culturally relevant health care provision, and education. Sharing circle participants discussed strengths and protective factors such as kinship, connection to culture, and spirituality. Indigenous patient navigation, inter-organization collaboration, and community relationship building were recommended to ameliorate barriers and bolster strengths. Recognizing barriers to access, coordination, culturally relevant health care provision, and education can further champion community strengths and protective factors and frame effective cancer care strategies and equitable cancer care for Indigenous people in Saskatchewan.

## 1. Introduction

Indigenous cultures are richly diverse across the globe, and yet similar colonial legacies have created disparities in health, poverty, and life expectancy even in economically developed countries (Australia, Canada, New Zealand, and the U.S) [1,2]. Although Indigenous Peoples represent a smaller percentage of the population, they often experience disproportionately worse health outcomes [2]. Cancer is now one of the leading causes of death in Native Americans and Alaskan Natives [3], Aboriginal and Torres Strait Islander People [4], Māori [5], and Indigenous Peoples of Canada (First Nations, Métis, and Inuit) [6,7], where studies report higher incidence rates, lower screening rates, and a greater likelihood of death after diagnosis [1,4,6,8]. For other Indigenous groups, such as the Sámi in Europe and Indigenous Peoples of Latin America, there are inadequate surveillance data; however, there is some evidence of small disparities in cancer incidence compared to the general population [9]. With the current knowledge, the higher rates of cancer diagnosis and mortality for Indigenous Peoples constitute a phenomenon that requires attention across continents [1].

First Nations and Métis Peoples in Canada have previously experienced lower rates of all cancer incidence and mortality compared to the general population [10]. However, in recent decades, a concerning trend has occurred among Indigenous patients, who now develop cancer at higher rates than previous generations [11]. Today, First Nations and Métis Peoples are impacted by higher rates of diagnosis and lower cancer survival rates across Canada [8,12]. In Ontario, First Nations people have higher incidence of certain cancers and overall higher mortality compared to the general population, with reports indicating that less than half will survive more than five years after diagnosis [6]. In Manitoba, data from 2004–2011 showed that First Nations people had much lower survival and later diagnosis than the rest of the population, and First Nations people living on reserve had later-stage diagnosis compared to those living off-reserve, indicating there are substantial barriers to health services and screening [13,14]. Saskatchewan has reported similar trends, with greater proportions of late-stage diagnosis for northern and First Nations populations compared to the southern half of the province [15]. Because cancer is now one of the top three causes of death for Indigenous people in Canada [16], effective, accessible, and culturally responsive cancer care strategies should be a priority.

Given that the Canadian health care system tends to be complex, fragmented, and multi-jurisdictional [17], experiences with cancer diagnosis, treatment, and aftercare may be especially difficult for Indigenous patients and families. These challenges also occur in the context of colonial policies and intergenerational trauma that are known to have considerable effects on health and well-being [18,19,20]. The suppression of cultural practice through colonial policies and the residential school system in Canada have contributed to long-lasting effects on well-being among Indigenous peoples [1]. This history has shaped broader social, cultural, political, and economic conditions that impact equitable health care access to present day. These conditions influence epidemiological trends in diseases including cancer [18]. Considering that access to health care, as well as culture, are recognized as determinants of health [21,22,23], employing culturally relevant care has recently become a more significant aspect of health care provision across Canada [20,24].

Research has shown that trust and world view are significant aspects of Indigenous experiences in the health care system [25,26], and that Indigenous cancer patients may prefer to incorporate traditional healing and cultural practices in the healing process [27]. Additionally, other studies have indicated that creating safe spaces to talk about diagnosis was often missing in the cancer support system [19]. Indigenous patients may experience marginalization in the health system, limited social support, or a reluctance to face social stigma around cancer diagnosis [17,19,28]. Factors such as limited local services, lack of education, and barriers to screening can be associated with later diagnosis in First Nations [13,19]. Although studies in Canada have investigated Indigenous-specific cancer care access [29], decision-making [30], epidemiology [31], and the role of spirituality during the cancer journey [28], current studies about Indigenous cancer care in Saskatchewan are either outdated or scarce [10,31]. Some research has examined Indigenous cancer patient experiences using sharing circle methods [25,32], or explored cancer care supports from service provider views [8]. Despite a concerning trend of higher cancer incidence and mortality among Indigenous peoples [29,33], there is limited understanding of cancer support for Indigenous patients during their cancer journey in Saskatchewan.

To address these limitations, championing strengths and protective factors can help frame effective cancer care strategies and support a continual movement towards equitable health. A strength-based approach has been described as a recent “paradigm shift” [34] within the fields of mental health, social work, psychology, and other public health professions [34,35]. This approach discourages a deficit-centered discourse and gives relevance to wellness in terms of elements outside of the individual, such as community, spirituality, and environment, a perspective shared by various Indigenous world views [34]. This approach opposes the narrative of disempowerment and positions the ‘problem’ as external to the individual, reducing blame and increasing awareness of solutions that may already exist [35]. Promoting the strengths and resilience among a population has relevance for First Nations and Métis people that experience intergenerational trauma from residential schools, loss of culture, and other acts of colonial policies within Canada. Adopting a strength-based approach for Indigenous cancer care could provide appropriate and effective health system strategies.

The purpose of the project was to examine and ameliorate gaps in cancer care support services using a strength-based approach. To do so, this article presents findings from a multi-method needs assessment entitled *Sâkipakâwin*, the Cree word for budding or sprouting. Guided by a patient-partner-led advisory committee to understand the support needs, both formal and informal, for First Nations and Métis people’s cancer care, we assessed cancer care support needs in Saskatchewan from the perspectives of health system leaders and Indigenous community members. Where possible, we strive to understand First Nations and Métis perspectives and avoid a pan-Indigenous approach, as we recognize each group faces unique barriers to cancer care provision. By detecting these gaps, we recommend culturally appropriate and patient-centered cancer care services for Indigenous cancer patients and families as they journey through the health system.

## 2. Materials and Methods

### 2.1. Study Design and Setting

Because Indigenous patients are the key knowledge holders, a patient-oriented research design was developed to foster relationships and equitable participation from Indigenous patients, families, and communities. In the spring of 2017, a research team of researchers and clinicians, led by Indigenous cancer patients, conducted a needs assessment for cancer supports in Saskatchewan. This study was part of a larger needs assessment that included an environmental scan of Indigenous-specific cancer supports in Canada [36] and service provider focus groups in Saskatchewan [8]. To recognize the principles of ownership, control, access, and possession (OCAP^®^) [37], a Memorandum of Understanding (MOU) was signed between the principal investigator and the First Nations communities to conduct the sharing circle. The consent agreement included non-disclosure of verbatim quotes, and thus quotes are paraphrased to uphold confidentiality of the community members and their identity but portray the nature of the original quote.

The project, including the development of the proposal for funding, was informed by five Indigenous patient and family partners. An advisory committee comprising these partners and representatives from the Saskatchewan Cancer Agency (SCA), Métis Nation Saskatchewan (MN-S), Northern Inter-Tribal Health Authority (NITHA), Saskatchewan Health Authority (SHA), and the University of Saskatchewan met a total of nine times to guide the project. Formal ethical approval was granted by the Behavioral Research Ethics Board (Beh-REB) at the University of Saskatchewan for the duration of the project.

### 2.2. Theoretical Approach

Given that this project champions the capacity of First Nations and Métis people as experts in their own cancer journeys, we adopted a strength-based lens for this work. This approach helps to illuminate ‘health-promoting’ motivations, behaviors, and protective factors to empower people and populations towards well-being to re-examine the context in which health research, policy, and practice can be applied [34,38,39]. Using this lens can re-orient solutions towards the strengths of First Nations and Métis people in Saskatchewan in light of generations of historical trauma from colonialism in Canada [34].

### 2.3. Data Collection

To understand health system leadership perspectives, key informant interviews were conducted with senior health care administrators or First Nations and Métis leaders between July and September 2018 and June and November 2019, respectively. Key informants were purposively selected by the advisory committee to represent senior health care and cancer care administrators. Upon receiving informed consent, audio-recorded interviews were conducted by telephone using a semi-structured interview guide with open-ended questions. Interviews lasted approximately 20 to 30 min and the questions aimed to identify what supports are available to Indigenous patients and their families, which are most important, what is needed, and what barriers exist for current services.

Following the key informant interviews, a sharing circle with patients and family members was facilitated to explore Indigenous cancer journeys. Sharing circle participants were recruited by health care workers from Indigenous communities in northern Saskatchewan by approaching those who were cancer patients or survivors or family members. The sharing circle method, an ancient Indigenous practice used to discuss community issues, was chosen as a culturally relevant space to discuss topics and to foster relationships and trust between participants and researchers [25,32]. After a formal agreement with a First Nations tribal council, individual consent for an audio recording of the sharing circle was obtained from each participant. The duration of the circle was approximately 1.5 h. An Elder was present at the gathering to open with prayer and provide support for the members during the session if necessary. Compensation was given in the form of refreshments and an honorarium.

### 2.4. Data Analysis

Key informant interviews were transcribed verbatim and analyzed using a deductive thematic analysis in NVivo (Version 12) to derive themes around supports and barriers. The analysis followed a five-step approach adapted from Braun and Clarke [40] by two researchers: (1) familiarization of the data, (2) independent generation of codes, (3) independent summarization of codes into themes, (4) reviewing of themes by both researchers, and (5) grouping formal themes by both researchers. Upon validation, adjustments were made to the original codebook.

Audio from the sharing circle was transcribed verbatim by the University of Saskatchewan Social Sciences Research Laboratories. Afterwards, transcripts were reviewed by two researchers, and the data were coded in NVivo (Version 12) in categories related to cancer diagnosis, cancer treatment, and after-care. A multi-stage deductive thematic analysis was conducted using the same five-stage process [40]. The analysis was guided by a strength-based lens, particularly in identifying positive experiences or supports and community-level strengths during the cancer journey.

Themes from key informant interviews were used to capture health system-level perspectives on the barriers to Indigenous cancer care, while the sharing circle, having no structured interview questions, explained community-level strengths relating to the cancer journey. By using these separate methods, we provide a picture of supports and barriers from patient, family, community, and system-level perspectives to inform Indigenous cancer care recommendations. These results were shared with the advisory committee for input, and final recommendations were discussed during a knowledge translation meeting in November of 2021.

## 3. Results

### 3.1. Sample Characteristics

A total of six key informant interviews were conducted with one senior cancer care administrator and five First Nations and Métis leaders and physicians. After a thematic analysis, we summarized results from the interviews into overarching themes around impacts and barriers. The recommendations from these findings are a result of a final meeting with the advisory committee.

A total of 18 participants (5 facilitators, 12 patients and family members, and one Elder) from six different communities in northern Saskatchewan participated in the sharing circle hosted in a First Nations community. Both female (*n* = 7) and male (*n* = 5) patients or family members participated from the 60–80-year-old age group. At the time of the sharing circle, a total of seven were survivors (*n* = 7), two were patients (*n* = 2), and three were family members (*n* = 3). Dates of diagnosis of patients or family members ranged from 1986 to 2018 for various cancer types, such as breast, colon, bone marrow, thyroid, and skin (squamous carcinoma).

### 3.2. Barriers to Existing Supports

We identified several social, geographic, economic, and organizational factors that prevent Indigenous cancer patients from accessing and receiving supports. These factors were summarized into broad themes from the key informant interviews (Table 1). Discussion from the sharing circle also echoed many of the same barriers presented below.

#### Impacts for Communities, Patients, and Families

Key informants, as physicians and individuals in health leadership positions, offered a valuable perspective of both system-level and patient-level experiences to examine key barriers to care. From these perspectives, it was clear that the physical remoteness from cancer treatments in city centers was a barrier to treatment. Since treatment requires travel from home communities, patients and families experience isolation from each other, and the familiarity of home is lost during the treatment process. Key informants emphasized the need for emotional support for the patient and family when travel is required for patients. Socioeconomic factors also impact patients’ access to care and decisions around receiving care. Although medical transport is covered by Indigenous Services Canada for First Nations patients with registered Indian status, there are still upfront costs that can influence the patients’ decision to continue treatment. As a result, certain impacts are felt at the very outset of health care provision for both patient and family.

Coordination of care was another prevalent barrier to assess and delivery of supports. A multi-jurisdictional health system creates a patchwork of coverage between provincial and federal districts which can cause confusion for both health providers and patients. Additionally, frequent rotation between oncologists and northern practitioners creates fragmentation and information breakdown. Language barriers, including using medical terminology for prognosis and treatment, can also create a disconnect between patient and providers. One key informant commented on how they often encountered uncertainty with their Indigenous cancer patients. Key informants also called attention to the limited data on Indigenous cancer patients at the health system level. A recurrent theme of the lack of navigation of services for patients was a key barrier described by key informants with their experience with patients in the health care system.

A few key informants commented on the difference between Indigenous and Western concepts of medicine, health, illness, and death. Some perceived that underappreciating these differences can create misunderstanding and judgement between patient and provider and is still a significant challenge to culturally relevant care. Likewise, experiences of racism create mistrust of the health care system and prevent patients from receiving dignified care. Key informants explained how some ambiguity around what culturally safe care looks like is a barrier itself, and that a pan-Indigenous approach is inadvisable. Traditional medicine, while chosen by some Indigenous cancer patients, is not well-supported in the Saskatchewan health care system and can require more recognition from doctors. At a system level, a shortage of Indigenous staff representation and limited data on Indigenous patients prevent what could support policy decisions. It was discussed how these contribute to patients feeling unseen while receiving care. While this is true, one informant commented on how there is perceived racism of their non-Indigenous colleagues when they are faced with system and skill limitations even when there is no ill intent. As one put it: “*it adds another barrier to compassionate care*”. This underscored the complexity of patient experiences and the reality of health system constraints impacting care at the same time. Overall, culturally safe and responsive services were perceived to be lacking in Saskatchewan.

We found a common theme regarding a lack of knowledge, from awareness of supports to the delivery of care and to patients’ understandings of diagnosis and treatment. Participants expressed a need for education at both the patient and health care provider level, as awareness and education are inextricably linked. Some discussed that limited education around differing Western and Indigenous world views will continue to prevent relationship building and understanding.

### 3.3. Strengths

Despite multifaceted barriers to care, the community sharing circle illuminated the inherent strengths and protective factors of First Nations patients and families. We discuss the broad themes of kinship, connection to culture, and spirituality in Table 2.

#### 3.3.1. Kinship

Stories from the sharing circle underscored the importance of support from spouses, significant others, and other relatives throughout the whole cancer journey. The experience of travelling away from family was stressful for some patients who recalled these moments, though when family was in the city, this made a considerable difference. Participants alluded to how travel companions provide valuable emotional and logistical support and accompany patients during their appointments. As a vital support, community members also acknowledged that caregivers require significant support themselves, given that experiences are shared and the burden is not only on the cancer patient.

#### 3.3.2. Connection to Culture

Some Indigenous patients commented on how they used traditional medicine and cultural practices to support their cancer journey. Community members discussed how they maintain and connect with cultural practices, such as smudging, during and after cancer treatment. Although traditional medicine is not well-supported during cancer care, many found the ability to use and benefit from it during their cancer journey. One participant recalled a trip to their doctor who saw the cancer improving, and the participant told the doctor they used traditional medicine. An Elder alluded to how the environment is an aspect of support and healing because of people’s relational connection to the land. Other themes around friendship and kinship intertwined with connection to culture as community cohesion is an aspect of cultural connection.

#### 3.3.3. Spirituality

Spirituality was identified as a significant element during the cancer journey by several community members. An Elder described the importance of prayer during sickness and healing, conveying a broader understanding and acknowledgement of health and healing. This also underlined some cultural differences between Western and Indigenous culture whereby broader determinants are less integrated in the Western biomedical model. The themes of kinship, connection to culture, and spirituality were found to be positive factors for patients during their cancer journey.

### 3.4. Recommendations

Informed by the key informant interviews and sharing circle discussions, the advisory committee emphasized actions required to create a more holistic and culturally safe experience for cancer patients to achieve Indigenous equity, inclusion, and dignity during the cancer journey. Given that First Nations and Métis communities are diverse in their experiences and histories, a pan-Indigenous approach is inadvisable. Therefore, the following recommendations have been summarized and then finalized by the patient family partners and organizational members of the advisory committee.

An Indigenous patient navigator and advocate was the most prominent recommendation. A navigator’s role would be to increase understanding and communication between patients and their health care providers, recognize the cultural needs of Indigenous people, translate to Indigenous languages, relay medical terminology and concepts, and guide patients towards the services that are available to them. Navigators and care counsellors are already offered for Indigenous cancer patients in provinces such as Alberta, British Columbia, and Ontario [41,42,43].

The lack of provincial and federal coordination of health services could be addressed by encouraging inter-organization collaboration and engaging in more relationship building at this level. An Indigenous cancer care strategy for Saskatchewan was recommended to (1) bridge gaps between services provided by these governments and organizations, (2) address the need for Indigenous navigation of the health care system including cultural awareness and humility, language translation, and medical language interpretation, and (3) recognize the complexity of Indigenous Sovereignty.

The advisory committee commented on the importance of working diligently with communities to build relationships and understand what is advised from their experience to avoid redundancy. The committee suggested to create strong connections among the organization to support navigators. Likewise, key informants suggested additional check-ins and follow-ups for patients and to use technology to our advantage as an effective way to address geographic and financial barriers. Doing so can identify and address problems early and determine whether patients can receive care in their community or if travel is required to the city. Overall, the advisory committee and key informants agreed that enhancing communication and education can be a solution.

Acknowledging cultural differences during the cancer care continuum, expanding community supports and services, and recognizing traditional medicine in the cancer journey are all elements in addressing the broader social and cultural barriers to health provision. Likewise, policy decisions that give First Nations and Métis people a greater voice can guide the administration towards more effective and holistic cancer strategies.

## 4. Discussion

While cancer care services for diagnosis, treatment, and follow-up are provided for all patients, regardless of ethnicity, there are barriers to equitable, safe, and respectful care for First Nations and Métis cancer patients. Most participants from the sharing circle and key informant interviews agreed that First Nations and Métis patients face challenges from the very outset of the cancer care continuum. When the cancer journey is complicated by fragmented and multi-jurisdictional cancer care, access to quality care is especially challenging for First Nations and Métis patients in Saskatchewan. Despite system-level barriers, communities continue to move through the system and experience challenge to cancer care. These barriers must be addressed to bolster the strength of communities and improve the quality of cancer supports.

This research highlights major geographic, cultural, social, economic, and system-level factors that influence cancer support services for First Nations and Métis patients. The findings present significant barriers related to access to care, coordination of care, lack of culturally relevant health care provision, and education. Although barriers such as socioeconomic factors, language, and remoteness are experienced by other populations in Canada [44], the layered social, cultural, and multi-jurisdictional factors for Indigenous patients make navigating the cancer journey especially difficult. Many of these themes are not unique to Saskatchewan, such as the gaps between provincial and jurisdictional health care systems which are known to influence health disparities [11,16]. The lack of services in communities [11], geographic barriers [16], inadequate navigation [16], and socioeconomic barriers to care [45] are common challenges across provinces [11]. As stated by the National Collaborating Centre of Indigenous Health (NCCIH), access to health services is a significant determinant of health for Indigenous Peoples in Canada [21].

As our findings have illustrated, broader determinants are intrinsically linked with patient-level experiences and are crucial components of patient access and provision of care. Akin to the social determinants of health are broader cultural determinants such as histories and cultural practices that shape Indigenous peoples’ relationships with the world, with health, and with their environment [23]. In Canada, the oppression of Indigenous peoples for generations has damaged not only their wellness, but also their relationship with health care systems [20]. As this study has shown, culturally appropriate care and cultural awareness are important factors, and many of the recommendations reflect these needs. Indigenous patient navigation was the main recommendation from the findings and advisory committee, and has been implemented in other provinces to ensure and augment patient-centered care [41,42,43]. Some studies have found that Indigenous patients can have limited opportunities to talk about cancer due to minimal supports and complex relationships with the health system [17,19], which further reinforces the need for culturally responsive care and the use of sharing circles as a method of research [17]. This suggests that proper acknowledgement and incorporation of distinct health care supports for Indigenous patients can have positive impacts on health. This challenges the idea that Indigenous patients are expected to ‘fit in’ to the health system, as Indigenous world views are often different from the Western medical health model [26]. Instead, culturally safe care offers a way to make space for both Indigenous and Western world views.

Despite these barriers, the strengths of Indigenous cancer patients have also been illuminated through our findings. Other studies have defined these as ‘protective factors’ [34,46] which help to achieve greater well-being. These strengths were categorized into three broad themes of kinship, connection to culture, and spirituality. Research acknowledges that kinship is a significant factor because of the emotional, practical, and decision-making support family members can provide [12]. The ability to incorporate traditional medicines is a unique aspect of Indigenous healing and can be a major part of the cancer journey [45]. Other studies have found spirituality to be a significant aspect of Indigenous women’s breast cancer journey in Saskatchewan [45], along with other cultural practices such as prayer, smudging, and sweat lodges during recovery [45]. Broader determinants of health, such as community, environment, culture, spirituality, and the interconnectivity of such factors, are also commonly represented in various Indigenous perspectives on wellness and well-being [47,48]. Disseminating strength-based approaches to health care is a necessary ‘paradigm shift’ [34] to support effective strategies by and for Indigenous peoples to improve health outcomes and experiences for cancer patients and their families.

To advance future efforts to support Indigenous health equity, the Truth and Reconciliation Commission (TRC) of Canada present distinct Calls to Action to follow [49]. Our recommendations address Call to Action #20, which acknowledges the distinct health needs of First Nations, Métis, and Inuit people, as opposed to overlooking their diversity as Peoples with unique histories and cultures [49,50]. Embedding culturally relevant care addresses Call #22 which recognizes the value of Indigenous healing practices when requested by Indigenous patients. Such practices have long been disregarded in Canadian medicine [36]. An Indigenous patient navigator would increase Indigenous staff representation, and providing cultural competency training for health care professionals would address Call #23 [49]. The advisory committee recommendation to establish an oversight committee and Indigenous Cancer Care Strategy could ensure these calls are championed.

### Strengths and Limitations

Our study has several limitations including the small sample sizes of the sharing circle and key informant participants. Therefore, it is possible that our findings may have introduced selection bias. However, we were still able to speak with system-level administrators of greatest relevance to the project, given that Saskatchewan has a single cancer agency and is a relatively small size for a province. Although we had engagement with relatively few participants, employing a design with Indigenous partners was a strength of the study. The sharing circle method was used as a culturally appropriate way to understand Indigenous perspectives and establish a relationship between researchers and community members [32]. Circles are significant in Indigenous culture, and sharing circles are a way to speak and listen without judgement or an agenda [32]. This design was used to support OCAP^®^ principles, given that trust and world view are significant factors in the experiences of Indigenous peoples with the health care system and in research [25].

This paper also reports only two aspects of the multi-phase project that included service provider interviews, and thus only presents the perspectives from two methods. We also recognize the plurality of Indigenous experiences and perspectives and the inability to capture everything from this project alone. However, recommendations from the advisory committee provided a voice from communities and Indigenous governments. Guidance from the advisory committee helped to uphold OCAP^®^ principles and a formal MOU agreement between researchers and community ensured a relationship from the outset of the project. The recommendations address the TRC Calls to Action and specific directives from the literature to engage Indigenous communities as part of the strategic process for cancer care reform [1].

Lastly, this study has a bias towards First Nations perspectives, with small representation from one Métis key informant and a small number of Métis members on the advisory committee. However, this project coincided with another project through MN-S who hosted consultation with 400 of its citizens [51]. This study found similar findings to ours, including limited access to care due to travel and financial burdens, and inadequate quality of care associated with lack of education, communication barriers, limited local supports and health professionals, and experiences of racism.

## 5. Conclusions

Our findings present the barriers to cancer care in Saskatchewan for Indigenous patients and families and amplify the strengths of existing supports. By using a strength-based approach, we provide urgent action areas and positive aspects of supports already existing within communities. This study also presents data from two different methods and incorporates community perspectives with recommendations guided by Indigenous partners and representatives. Given the inequities of cancer outcomes in Indigenous populations globally, there is a need for better preparation for an increasing number of Indigenous cancer patients and to ensure cancer care systems can provide culturally responsive care and education. Applying these findings will help to frame effective strategies and better support Indigenous cancer patients along their cancer journeys.

## Figures and Tables

**Table 1:** Key Informant Perceptions of Barriers to Existing Cancer Supports.

Theme	Determinant	Exemplar Quotes
Access to Care	Cancer treatments only available in cities and little to no local treatment Remoteness and isolation Travel and accommodation Socioeconomic and precarious finances Racism and misunderstanding	*“You might be by yourself, you might not have any family support being able to come with you, because the way the funding is provided. So, often that distance from the remote northern place to go for the treatments [is a challenge]”.* *“It’s sometimes very challenging for people to access care even though they live right here in the middle of Saskatoon”*. *“I think people are falling through the cracks quite a bit. [We need to be] ensuring that people are comfortable. Making sure that they’re connected with things like palliative care, or homecare”.* *“Sometimes inefficiencies in our system and limitations in our abilities as health providers, and the limitations of Western medical model are deemed racist… sometimes [my colleagues] are unfairly judged, and the limitations of their skillset or the care that we provide are deemed a cultural issue, when in fact they aren’t”.*
Coordination of Care	Fragmented care Limited services in communities Communication between patients and health care providers Language barriers Lack of navigation through cancer care	*“[Another point worth sharing is] working with Health Canada or Indigenous Service Canada, to be able to address jurisdictional challenges, from a Federal perspective. There’s lack of coordination between Federal and Provincial funded health services, there’s no relationship in that area. So there’s [got to] be a mechanism to allow the jurisdiction challenges to be addressed within that system [of the] Cancer Agency”.* *“Language is an issue. Particularly in the north, people come down to an urban center such as Saskatoon and they are not familiar with even the terminology that is used through the continuum of cancer care, let alone not being able to communicate in their own language. That’s the problem”.*
Lack of Culturally Relevant Health Care Provision	Disparities between Western and Indigenous world views Traditional medicine not well supported Lack of data systems for Indigenous patients Absence of Indigenous staff	*“There are cultural differences, and people don’t really appreciate or understand that. They feel that our Indigenous peoples should just fit in to the existing system, and they don’t. They never have, and that’s why there’s so much health disparity. I think the idea that we just keep doing the same for everybody is a big barrier”.* *“Right from governance; there’s lack of understanding around culturally appropriate practice; from a senior leadership perspective, very little if any, around relationship knowledge of culturally appropriate programming. So, from a governance and operational level, there is none”.* *“…there’s very little, if any, supports that are culturally appropriate programming or services that support Indigenous patients that are coming into the Cancer Institution”.*
Education	Lack of education during prognosis and treatment Lack of education on how to provide culturally safe care Lack of acknowledgement of cultural differences and barriers	*“The idea of access and education. They go hand-in-hand, they’re one in the same. I would say that that’s the biggest thing”.* *“Explaining some of the anatomy, supporting their understanding, and reassuring when you can reassure is a huge thing for [cancer] patients”.* *“Part of that challenge is the lack of understanding of what culturally appropriate services are actually needed. There needs to be relationships with the communities, to engage to them and get a better understanding of what the community needs [around] culturally appropriate service and adequate programs that will suffice the Indigenous community, the First Nation, M* *étis”.* *“If there’s medication that’s required, will it be covered, will it not be covered?… Do you need to take it with food, without food? If you are somebody who doesn’t necessarily have food all the time, that can be a challenge. It might seem like something simple, but [it can be] very difficult…I think there’s a lot of judgements are passed, that are based one’s own perception of what they might do. Not understanding what some of the challenges and barriers are that many of our Indigenous peoples are facing”.*

**Table 2:** Strengths and informal supports identified in sharing circle.

Theme	Paraphrased Quotes From Participants
Kinship	*“My wife always supported me”* *“I wish there were better ways to help a supporter. It is a stressful time, and we are going through a similar experience”*
Connection to Culture and Traditional Healing	*“The land offers us medicine”* *“I would go to the bush with a friend each summer, they help me. I am trying to find my own medicines”.*
Spirituality	*“The one up there will heal us”*

## Data Availability

The data from these findings are not publicly available and cannot be provided by the corresponding author, as this would compromise the confidentiality agreements with the participants in the study.
